# Reinterpretation of tuberculate cervical vertebrae of Eocene birds as an exceptional anti‐predator adaptation against the mammalian craniocervical killing bite

**DOI:** 10.1111/joa.13980

**Published:** 2023-11-22

**Authors:** Gerald Mayr, Ursula B. Göhlich, Zbyněk Roček, Alfred Lemierre, Viola Winkler, Georgios L. Georgalis

**Affiliations:** ^1^ Ornithological Section, Senckenberg Research Institute and Natural History Museum Frankfurt Frankfurt am Main Germany; ^2^ Geological‐Paleontological Department, Natural History Museum Vienna Vienna Austria; ^3^ Department of Palaeobiology Institute of Geology, Czech Academy of Sciences Prague 6 Czech Republic; ^4^ CR2P—Centre de recherche en Paléontologie–CNRS/MNHN/Sorbonnes Université, Bâtiment de Géologie Paris France; ^5^ Central Research Laboratories, Natural History Museum Vienna Vienna Austria; ^6^ Institute of Systematics and Evolution of Animals, Polish Academy of Sciences Kraków Poland

**Keywords:** adaptation, Aves, evolution, fossil birds, morphology

## Abstract

We report avian cervical vertebrae from the Quercy fissure fillings in France, which are densely covered with villi‐like tubercles. Two of these vertebrae stem from a late Eocene site, another lacks exact stratigraphic data. Similar cervical vertebrae occur in avian species from Eocene fossils sites in Germany and the United Kingdom, but the new fossils are the only three‐dimensionally preserved vertebrae with pronounced surface sculpturing. So far, the evolutionary significance of this highly bizarre morphology, which is unknown from extant birds, remained elusive, and even a pathological origin was considered. We note the occurrence of similar structures on the skull of the extant African rodent *Lophiomys* and detail that the tubercles represent true osteological features and characterize a distinctive clade of Eocene birds (Perplexicervicidae). Micro‐computed tomography (μCT) shows the tubercles to be associated with osteosclerosis of the cervical vertebrae, which have a very thick cortex and much fewer trabecles and pneumatic spaces than the cervicals of most extant birds aside from some specialized divers. This unusual morphology is likely to have served for strengthening the vertebral spine in the neck region, and we hypothesize that it represents an anti‐predator adaptation against the craniocervical killing bite (“neck bite”) that evolved in some groups of mammalian predators. Tuberculate vertebrae are only known from the Eocene of Central Europe, which featured a low predation pressure on birds during that geological epoch, as is evidenced by high numbers of flightless avian species. Strengthening of the cranialmost neck vertebrae would have mitigated attacks by smaller predators with weak bite forces, and we interpret these vertebral specializations as the first evidence of “internal bony armor” in birds.

## Graphical abstract

We report avian cervical vertebrae from the late Eocene Phosphorites du Quercy in France that are densely covered with tubercles. Micro‐computed tomography shows these fossils to have a remarkably thick cortex and dense interior. We hypothesize that this unique vertebral morphology served to strengthen the cervical column in the upper part of the neck and represents an anti‐predator adaptation against the mammalian craniocervical killing bite (“neck bite”).

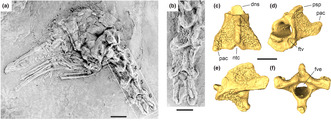

## 1 INTRODUCTION

The vertebrate fossil record shows an occasional occurrence of unusual osteological features. Most of these either represent extravagant morphological specializations (e.g., Szyndlar & Georgalis, 2023; Tapanila et al., 2013) or are of pathological origin (e.g., Schlüter et al., 1992). However, a few defy a straightforward explanation, and this is particularly true for avian cervical vertebrae that are densely covered with tubercles.

For the first time, these tuberculate cervical vertebrae were reported in a bird from the latest early or earliest middle Eocene (48 million years ago [Ma]) of Messel in Germany (Peters, 1995), which is currently known as *Dynamopterus tuberculatus* (Mayr, 2022). The only specimen of this large‐sized and possibly flightless species is a nearly complete skeleton on a slab, which exhibits numerous tubercles on the surfaces of the cervical vertebrae. These structures were considered to be a true morphological feature of the species, which was assigned to the cariamiform Idiornithidae in the original description (Peters, 1995).

**FIGURE 1:**
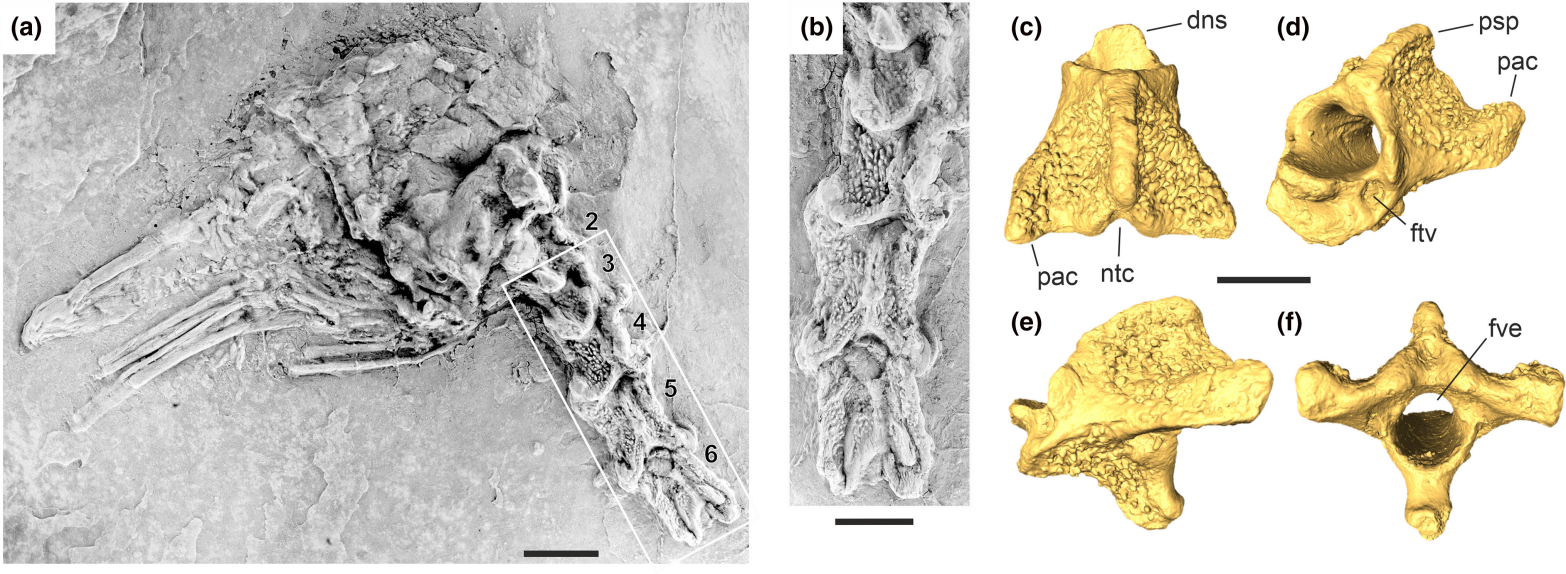
(a) skull and (b) cranialmost cervical vertebrae (detail of the framed area in (a)) of *Perplexicervix microcephalon* from Messel (SMF‐ME 11211a); coated with ammonium chloride, the vertebrae are numbered. (c–f) μCT‐based surface reconstructions of the newly identified avian axis MHNT.PAL.2020.0.36.13 from the site La Bouffie of the Quercy fissure fillings in (c) dorsal, (d) craniolateral, (e) lateral, and (f) caudal view. dns, dens; ftv, foramen transversarium; fve, foramen vertebrale; ntc, notch; psp, processus spinosus; pac, processus articularis caudalis. The scale bars equal 5 mm.

Similar tuberculate cervical vertebrae were subsequently described from a much smaller avian species from Messel, which was named *Perplexicervix microcephalon* (Figure 1a,b; Mayr, 2007, 2010). It was hypothesized that the tubercles represent a pathological condition (Mayr, 2007), but this assumption was challenged by the recognition of similar structures in multiple further individuals of *P. microcephalon*, virtually all of which exhibit tuberculate vertebrae (Mayr, 2010).

Another species of *Perplexicervix*, *P. paucituberculata*, was recently identified in the early Eocene (53 Ma) British London Clay (Mayr et al., 2023). The surfaces of some cervical vertebrae of this species are covered with barb‐like structures that are smaller than the tubercles of the species from Messel. The holotype of *P. paucituberculata* mainly consists of a series of vertebrae, but postcranial bones that were tentatively referred to the species clearly differ from those of cariamiform birds and show a resemblance to the Otidiformes (bustards). Owing to its osteological distinctness, *Perplexicervix* was placed in a new higher‐level taxon Perplexicervicidae (Mayr et al., 2023).

One impediment of previous studies was the lack of histological data for these vertebrae, most of which occurred in flattened fossils on slabs. However, one isolated tuberculate cervical vertebra from the late Eocene (37–38 Ma) locality La Bouffie of the Phosphorites du Quercy in France was previously figured but remained unstudied (Mayr, 2007). Here we describe three further such vertebrae from these fissure fillings, two of which also come from the locality of La Bouffie. Altogether four cervical vertebrae with tuberculate bone surfaces are now known from the Quercy fissure fillings, which are distributed over four institutions, were independently collected within a timespan of several decades, and almost certainly stem from different individuals. These specimens are the only three‐dimensionally preserved vertebrae with pronounced surface sculpturing, and for the first time, we were able to perform micro‐computed tomography (μCT) imaging, which led to a new hypothesis on the morphology and possible evolutionary significance of these unusual structures that have no analog among extant birds.

## 2 MATERIALS AND METHODS

The fossils stem from the collections of the Natural History Museum Vienna, Austria (NHMW), the Institut des Sciences de l'Evolution, Université de Montpellier (UM), the Muséum d'Histoire Naturelle de Toulouse, France (MHNT), the National Museums Scotland, Edinburgh, UK (NMS), and the Senckenberg Research Institute Frankfurt, Germany (SMF).

Three of the cervical vertebrae with tuberculate surfaces from the Quercy fissure filings—the Toulouse specimen MHNT.PAL.2020.0.36.13, the Montpellier specimen UM BFI 3101, as well as an uncatalogued specimen in the collection of the Université Claude Bernard Lyon 1, France (Mayr, 2007)—are from the late Eocene locality La Bouffie. One vertebra, the Vienna specimen NHMW 2019/0059/0013, is from the old Quercy collections and lacks precise locality data. This vertebra was acquired in 1888/89 from A. Rossignol, Lacapelle‐Livron, and was associated with numerous isolated bones of amphibians and squamates, which were recently studied and likewise lack exact locality data (Georgalis et al., 2023; Georgalis, Čerňanský, & Klembara, 2021; Georgalis, Rabi, & Smith, 2021). The colour of NHMW 2019/0059/0013 is different from that of the La Bouffie specimens, indicating disparate diagenetic environments. Most likely, therefore, the Vienna specimen is not from La Bouffie, but from a different (albeit unknown) site of the Phosphorites du Quercy, which span a time interval from the middle Eocene to the early Miocene, with the majority of localities ranging between the late middle Eocene and the late Oligocene (Georgalis, Čerňanský, & Klembara, 2021; Mourer‐Chauviré, 2006; Pélissié et al., 2021).

High‐resolution microtomography (μCT) of two vertebrae (MHNT.PAL.2020.0.36.13 and NHMW 2019/0059/0013) and the skull of the extant rodent *Lophiomys imhausi* were conducted at the MRI platform of the Institut des Sciences de l'Evolution de Montpellier (UM) and the μCT facilities of NHMW and SMF.

## 3 RESULTS

**FIGURE 2:**
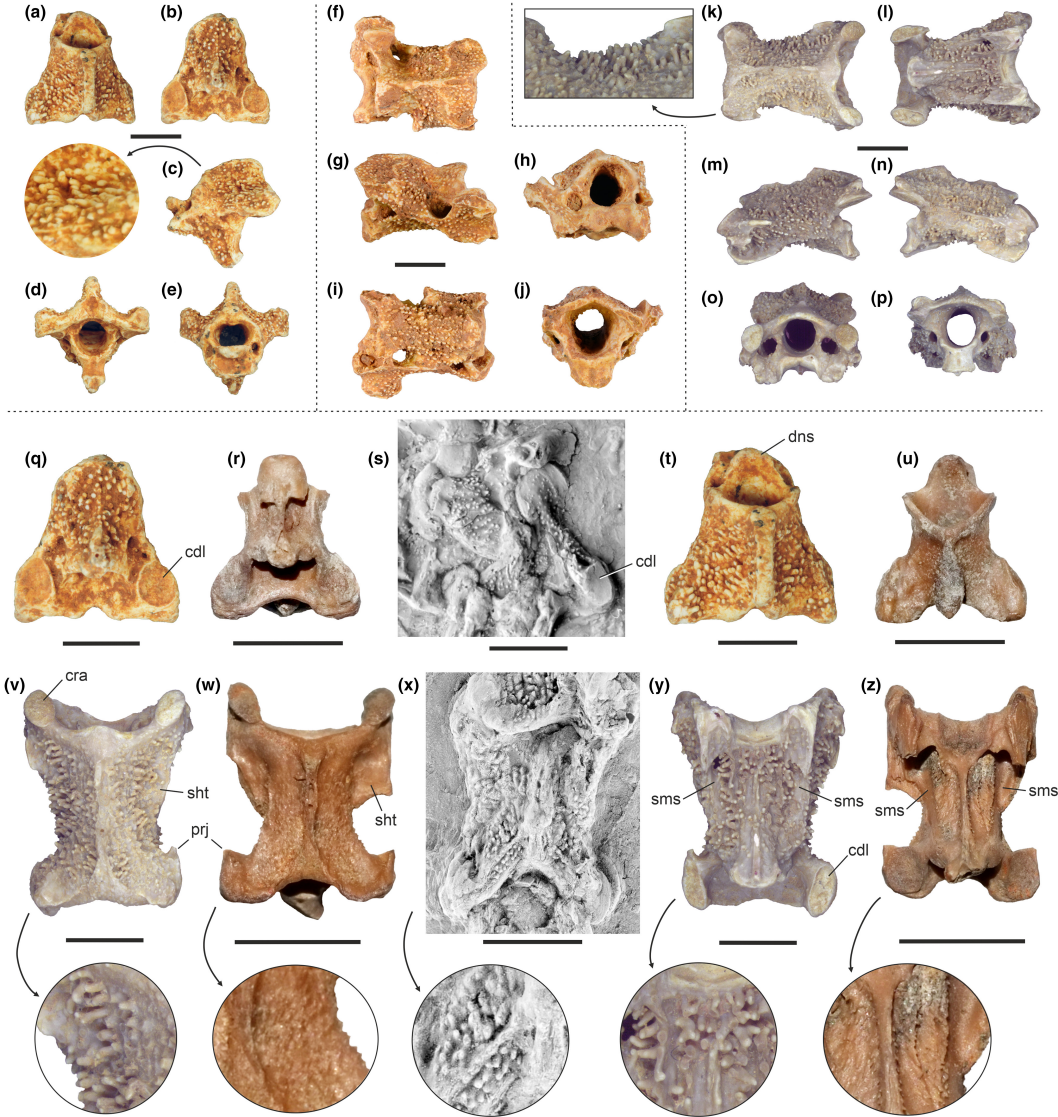
(a–p), (v), (y) the new cervical vertebrae with tuberculate surfaces from the Quercy fissure fillings in comparison to (s), (x) vertebrae of *Perplexicervix microcephalon* from Messel and (r), (u), (w), (z) *P. paucituberculata* from the London Clay. (a–e) MHNT.PAL.2020.0.36.13, axis in (a) dorsal, (b) ventral, (c) left lateral, (d) caudal, and (e) cranial view. (f–j) UM BFI 3101, third cervical vertebra in (f) dorsal, (g) right lateral, (h) cranial, (i) ventral, and (j) caudal view. (k–p) NHMW 2019/0059/0013, fifth or sixth cervical vertebra in (k) dorsal, (l) ventral, (m) left and (n) right lateral, (o) cranial, and (p) caudal view. (q), (t) MHNT.PAL.2020.0.36.13, axis in (q) dorsal and (t) ventral view. (r), (u) axis of *P. paucituberculata* (NMS.Z.2021.40.7) in (r) dorsal and (u) ventral view. (s) axis of *P. microcephalon* (SMF‐ME 3548) in ventral view; coated with ammonium chloride. (v), (y) NHMW 2019/0059/0013, fifth or sixth cervical vertebra in (v) dorsal and (y) ventral view. (w), (z) fifth or sixth cervical vertebra of *P. paucituberculata* (NMS.Z.2021.40.7) in (w) dorsal and (z) ventral view. (x) fifth cervical vertebra of *P. microcephalon* (SMF‐ME 11211a) in dorsal view; coated with ammonium chloride. The arrows indicate enlarged details of the tuberculate surfaces. cdl, caudal articulation facet; cra, cranial articulation facet; dns, dens; prj, cranially directed projection of processus articularis caudalis; sht, sheet‐like expansion of lateral portion of vertebral body; sms, smooth vertebral surface that guided vessels and nerves that passed through the foramen transversarium. The scale bars equal 5 mm.

The Toulouse specimen (Figures 1c–f, 2a–e; MHNT.PAL.2020.0.36.13) is an axis, which apart from its larger size resembles the axis of *Perplexicervix paucituberculata* from the early Eocene London Clay (Figure 2r,u; Mayr et al., 2023). As in this species, the dens is long and mediolaterally wide, but in the late Eocene Quercy vertebra, the processus spinosus is proportionally smaller and not as strongly dorsally protruding, and the notch between the processus articulares caudales (Figure 1c) is narrower and deeper. The Montpellier specimen (Figure 2f–j; UM BFI 3101) exhibits an osseous bridge, which delimits a lateral foramen and identifies the vertebra as the third or fourth cervical. The Vienna specimen (Figure 2k–p; NHMW 2019/0059/0013) is a fifth or sixth cervical vertebra and resembles a vertebra of *P. paucituberculata* from the London Clay (Figure 2w,z), which was initially identified as the third cervical vertebra (Mayr et al., 2023), but is now considered to also be the fifth or sixth cervical.

The axis MHNT.PAL.2020.0.36.13 in particular is much more densely covered with tubercles than that of *P. paucituberculata* from the London Clay. In the Quercy vertebrae, the surface structures are also more pronounced than in the vertebrae from the London Clay, and rather than being tubercles they are markedly elongate and have a villi‐like appearance, which is particularly evident in the Vienna specimen. As far as comparisons are possible, they correspond well the surface structures of the vertebrae of *P. microcephalon* and “*Dynamopterus*” *tuberculatus* from Messel.

**FIGURE 3:**
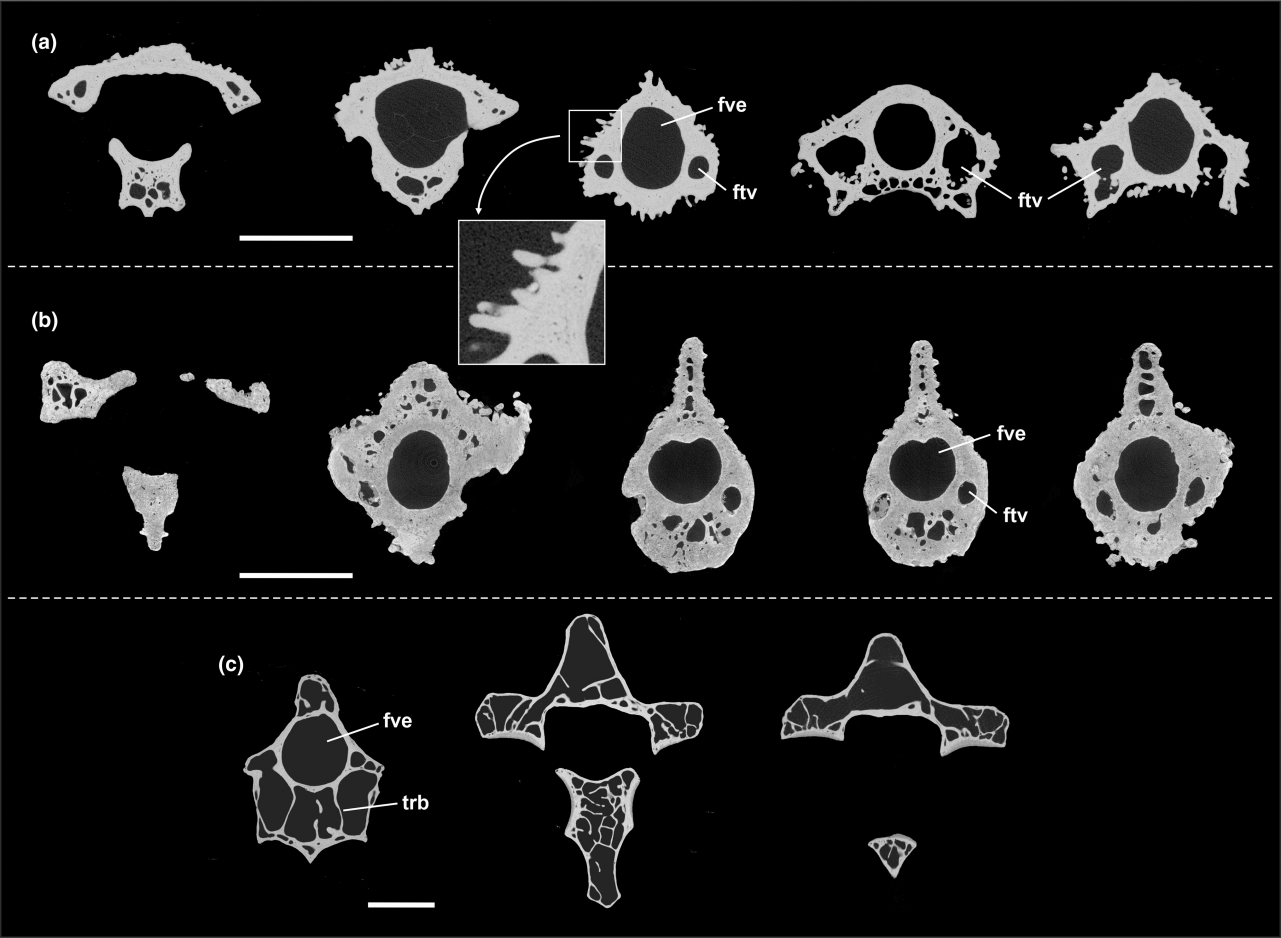
μCT scans of transverse sections of (a), (b) the fossil vertebrae from the Quercy fissure fillings (a: the fifth or sixth cervical vertebra NHMW 2019/0059/0013; b: the axis MHNT.PAL.2020.0.36.13) and (c) the axis of the extant Mute Swan, *Cygnus olor* (NHMW 4.896). ftv, foramen transversarium; fve, foramen vertebrale; trb, trabecle. The scale bars equal 5 mm.

In all three cervical vertebrae, the tubercles/villi have a symmetrical distribution on the left and right sides of the vertebrae. They cover most of the external vertebral surfaces, but are absent from the articular surfaces and the dorsal ridge of the processus spinosus. Tubercles are also absent from the caudoventral portion of the corpus and the attachment sites of intervertebral ligaments. In the NHMW specimen, there are a few tubercles within the foramina transversaria (Figure 3a). Well‐developed tubercles are furthermore largely absent from those areas on the ventral surface of the corpus, where vessels and nerves ran that passed through the foramina transversaria (the smooth, “slide‐like” vertebral surface is particularly evident in the vertebrae from the London Clay, but can also be observed in the Quercy specimens; Figure 2y,z).

At least some of the tubercles appear to be strung in longitudinal, craniocaudally extending rows. Closely adjacent tubercles usually are congruently aligned. Whereas most tubercles/villi direct more or less perpendicular to the vertebral surface, those on the vertebral arch of the axis point craniolaterally (Figure 2c). In the NHMW specimen, many tubercles are broken and it can be discerned that they are solid structures with a well‐differentiated cortex.

μCT scans were conducted of the MHNT and NHMW specimens (Figure 3a,b). Unexpectedly, these scans reveal a very thick bone cortex and an unusually dense interior of the bones, which exhibits much fewer pneumatic spaces and trabecles than the cervical vertebrae of most extant birds (Figure 3c; an exception are some specialized divers, see discussion). The scans furthermore show the tubercles to be outgrowths of the vertebral cortex and confirm the presence of some tubercles within the foramina transversaria.

## 4 DISCUSSION

Skeletons of *Perplexicervix microcephalon* and “*Dynamopterus*” *tuberculatus* show the tubercles to be mainly restricted to the cervical vertebrae, and all of the known *P. microcephalon* specimens with sufficiently well‐preserved cervical vertebrae exhibit a tuberculate surface of the vertebral cortex (Mayr, 2007, 2010; Peters, 1995). The wide occurrence of this feature in multiple individuals and its restricted distribution within the skeleton challenge a pathological origin. The μCT scans furthermore reveal that the tubercles/villi cannot be delimited from the bone cortex, which also conflicts with a pathological origin.

Another argument against a pathological origin comes from the different morphologies of the surface structures in the fossils from Messel and Quercy on the one hand and the London Clay on the other. As detailed above, their development is less pronounced in the older fossils from the London Clay, in which they are not differentiated into elongated villi‐like structures but form smaller and more barb‐like excrescences of the vertebral surface (Figure 2w,z). Finally, a pathological origin of the tubercles is contradicted by the fact that they are absent from functionally critical structures, such as the articular surfaces, the foramen vertebrale, and the vertebral surfaces that were in contact with the vessels leading into the foramina transversaria.

**FIGURE 4:**
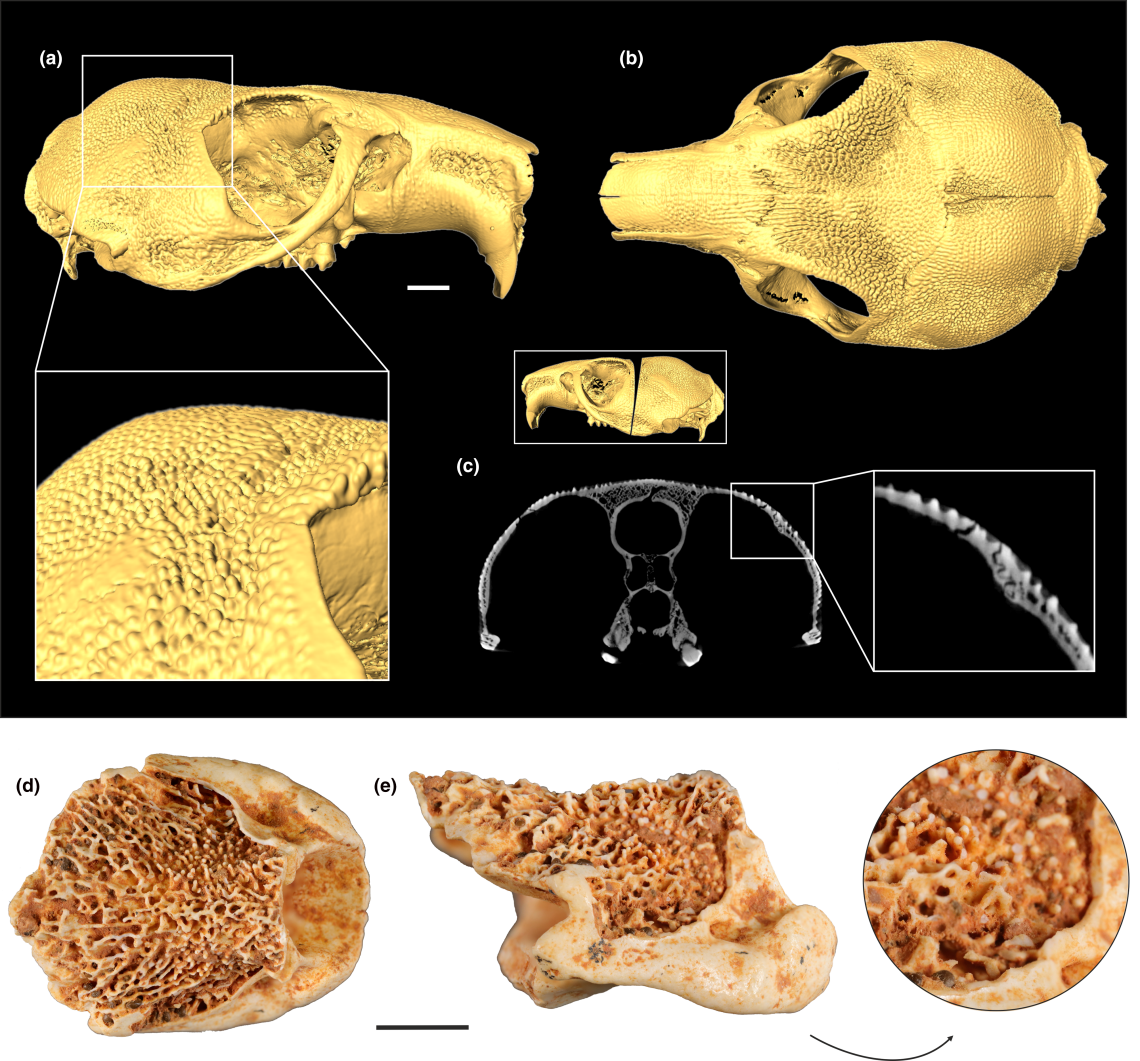
(a, b) μCT‐based surface reconstructions of the skull of the extant rodent *Lophiomys imhausi* (SMF 34609) in (a) lateral and (b) dorsal view, with an enlarged detailed of the sculptured surface. (c) cross section through the skull in the area shown in the framed inset, with an enlarged detail of the tuberculate surface. (d, e) partial axis of an undetermined mammal from La Bouffie (UM BFI 3102) in (d) dorsal and (e) lateral view; the arrow denotes a detail of the trabecles. The scale bars equal 5 mm.

That similar tubercles can develop through normal developmental pathways is shown by the skull of an unusual extant African rodent, the Maned Rat *Lophiomys imhausi*, which bears a tuberculate sculpturing in its caudal portion (Figure 4a–c; Kingdon et al., 2012; Lazagabaster et al., 2021: Figure 2). There is also a damaged axis of an undetermined mammal from La Bouffie, in which parts of the cortex are broken so that the interior of the vertebral corpus can be seen. In this specimen, the exposed trabecles likewise show a close resemblance to the tubercles on the external surface of the avian vertebrae (Figure 4d,e). We therefore hypothesize that the tubercles represent a true morphological feature, which gradually evolved over time and under some selection pressure.

All avian cervical vertebrae from the Quercy fissure fillings have matching sizes and are probably from the same or closely related species. Even though it is difficult to reliably identify early Cenozoic birds based on cervical vertebrae alone, the similarities between the Quercy vertebrae and those of *Perplexicervix*, which in the case of the fifth or sixth cervical include a cranially directed projection of processus articularis caudalis and a sheet‐like expansion of lateral portion of vertebral body (Figure 2v,w), suggest close affinities. We therefore hypothesize that the tuberculate cervical vertebrae characterize a distinctive clade of Eocene birds, for which the name Perplexicervicidae is available (Mayr et al., 2023). “*Dynamopterus*” *tuberculatus* is more likely to be a flightless representative of this clade rather than belonging to the cariamiform taxon *Dynamopterus*.

The μCT scans reveal that the tuberculate vertebrae have a thick cortex and very dense bone, whereas the cervical vertebrae of most extant birds exhibit large pneumatic spaces separated by narrow bony trabecles (e.g., Fajardo et al., 2007; Gutzwiller et al., 2013). In extant birds, unusually dense (osteosclerotic or pachyostotic) bone occurs only in some diving taxa, in which it contributes to a reduced buoyancy (Gutzwiller et al., 2013). The avifauna of La Bouffie does not include aquatic birds (Mourer‐Chauviré, 2006) and the flightless “*Dynamopterus*” *tuberculatus* as well as the fairly long‐legged, volant *Perplexicervix microcephalon* certainly had a terrestrial ecology. Hence, the thick cortex and dense bone structure of the cervical vertebrae from the Quercy fissure fillings most likely evolved to increase the mechanical strength of the bones.

The density and distribution of the trabecular bone of a vertebra is a result of the mechanical loading acting on it (Smit et al., 1997), so that the tubercles and the dense interior of the bones may have developed due to high forces exerted by the neck muscles. A correlation with muscular forces is suggested by the fact that many of the tubercles on the dorsal surface of the axis vertebra direct cranially, in the direction of contraction of the muscles attaching to this part of the vertebrae. However, the tubercles on the more caudal cervical vertebrae direct perpendicular to the vertebral body and, as noted above, there are also some tubercles within the foramina transversaria, which do not encompass muscles but conduct blood vessels and nerves. Possibly, therefore, the tubercles are a morphological corollary of developmental or functional constraints of the increased thickness of the vertebral cortex.

A functional interpretation of the tuberculate vertebrae is impeded by the fact that similar structures are unknown from extant vertebrates. Even though the vertebrae of some amphibians (e.g., the early to mid Cenozoic salamander *Chelotriton*; Böhme, 2008; Ivanov, 2008; Roček, 2019), one taxon of snakes (the extant *Xenopholis*; Jansen et al., 2009), and the extant chamaeleonid lizard *Brookesia* (Molnar & Watanabe, 2023: Figure 1a) exhibit granular surfaces, these are restricted to the neural spines or other parts of the dorsal surface of the vertebrae, with the only exception being the Cretaceous–Paleogene salamander *Piceoerpeton* and the Late Cretaceous pipid frog *Pachycentrata*, which also show some sculpturing in the ventral part of some vertebrae (Báez & Rage, 1998; Gardner, 2012). More similar to the surface structures of the fossil avian cervical vertebrae are those on the skull of the anuran taxa *Latonia* (Roček, 1994), *Pelobates* (Roček et al., 2014; Syromyatnikova, 2019), *Scaphiopus* (Roček, 1981), the Eocene *Thaumastosaurus* (Georgalis et al., 2023), the Late Cretaceous *Baurubatrachus santosdoroi* (Muzzopappa, 2022), and some gekkotan lizards (Glynne et al., 2020). Birds and most mammals do not show similar excrescences, the only notable exception being the above‐mentioned rodent *Lophiomys imhausi* (Figure 4a–c; Kingdon et al., 2012).

*Lophiomys* is the only mammal that is known to impregnate parts of its fur with plant toxins, and its unusual skull sculpturing in the back of the neck was interpreted as extra shielding of the brain that evolved in response to predation pressure (Kingdon et al., 2012). The cervical vertebrae of birds conduct and protect vital structures, that is, major arteries, nerves, and the spinal cord, and we consider it possible that the specialized morphologies of the Eocene species likewise evolved as an anti‐predator adaptations to mitigate attacks against the neck.

It is a distinctive feature of some mammalian predators to dispatch prey with a craniocervical killing bite in the neck or caudal portion of the skull. This behaviour evolved independently within carnivorans, primates, insectivores, and marsupials but is not found in other mammalian predators and reptiles, which dispatch their prey with undirected bites (Eisenberg & Leyhausen, 1972; Leyhausen, 1965; Steklis & King, 1978). The craniocervical killing bite is also employed to kill birds (Cuthbert, 2003; Lyver, 2000; Ratz et al., 1999), and with their presumed terrestrial ecology and fairly long necks, perplexicervicids are likely to have been prone to attacks by mammalian predators. We hypothesize that the surface tubercles and the thick cortex and dense interior of the vertebrae strengthened the vertebral spine in the neck region. In combination with behavioural anti‐predator adaptations, such as death feigning (which is known from some extant birds; Sargeant & Eberhardt, 1975), these vertebral specializations would have raised the survival rates of birds in attacks against the neck by small‐sized predators with comparatively weak bite forces.

In the earliest Cenozoic, Europe was geographically largely isolated from other continents and featured a high number of flightless birds, which indicates a low predation pressure (Mayr, 2022). During the Eocene, the extinct clade Hyaenodonta dominated in Europe, and modern‐type carnivorans first dispersed into Europe at the Eocene–Oligocene boundary, including the Feliformia (cats, mongooses, and allies) and Caniformia (weasels, dogs, and allies) (Solé et al., 2022), which are among the main mammalian predators of adult birds in many extant ecosystems (e.g., Hilton & Cuthbert, 2010; O'Donnell et al., 2015). It has been hypothesized that this faunal exchange terminated the existence of flightless birds in continental Europe (Mayr, 2022), and the immigration of more versatile mammalian carnivores may as well have led to the extinction of *Perplexicervix*‐like birds. This evolutionary scenario explains why tuberculate vertebrae are only known from the Eocene of Europe and can be falsified by the discovery of tuberculate vertebrae in birds from post‐Eocene strata or from fossil sites outside Europe.

Morphological anti‐predator adaptations are widespread among terrestrial vertebrates and include caudal autotomy in lizards or dermal armour in various groups of squamates and a few mammals (Broeckhoven et al., 2015). The tuberculate surfaces of the Eocene cervical vertebrae resemble the sculpturing of the osteoderms of some squamates (e.g., Buffrénil et al., 2011), and if our hypothesis is correct, they would represent the first evidence of “internal bony armor” in birds.

## AUTHOR CONTRIBUTIONS

GM conceived and designed the study, analysed and interpreted the data, prepared the figures, authored the first draft, reviewed subsequential drafts, and approved the final manuscript. GG identified the Quercy vertebrae, interpreted data, co‐authored the first draft, reviewed subsequential drafts, and approved of the final manuscript. VW performed CT scans of the Vienna vertebra, reviewed manuscript drafts, and approved the final manuscript. UBG, ZR, and AL contributed data, assisted with their interpretation, reviewed manuscript drafts, and approved the final manuscript.

## Data Availability

The data of all scans are curated by the institutions, in which the specimens are deposited; access can be requested through each institution. MicroCT scans of the axis of *Cygnus olor* (https://doi.org/10.57756/tw1z4s) and the fossil vertebra NHMW 2019/0059/0013 (https://doi.org/10.57756/fbf5wk) are available online. Other data supporting the findings of this study are available from the corresponding author upon reasonable request.
